# Cytotoxicity of polymers intended for the extrusion-based additive manufacturing of surgical guides

**DOI:** 10.1038/s41598-022-11426-y

**Published:** 2022-05-05

**Authors:** Felix Burkhardt, Benedikt C. Spies, Christian Wesemann, Carl G. Schirmeister, Erik H. Licht, Florian Beuer, Thorsten Steinberg, Stefano Pieralli

**Affiliations:** 1 Department of Prosthetic Dentistry, Faculty of Medicine, Medical Center, Center for Dental Medicine, University of Freiburg, Hugstetter Straße 55, 79106 Freiburg, Germany; 2 Freiburg Materials Research Center FMF and Institute for Macromolecular Chemistry, Albert-Ludwigs-University Freiburg, Stefan-Meier-Str. 21, 79104 Freiburg, Germany; 3 Basell Sales & Marketing B.V., LyondellBasell Industries, Industriepark Höchst, 65926 Frankfurt, Germany; 4 Department of Prosthodontics, Geriatric Dentistry and Craniomandibular Disorders, Charité -Universitätsmedizin Berlin, corporate member of Freie Universität Berlin, Humboldt-Universität Zu Berlin and Berlin Institute of Health, Assmanshauser Str. 4-6, 14197 Berlin, Germany; 5 Division of Oral Biotechnology, Faculty of Medicine, Medical Center, Center for Dental Medicine, University of Freiburg, Hugstetter Straße 55, 79106 Freiburg, Germany

**Keywords:** Cell biology, Medical research

## Abstract

Extrusion-based printing enables simplified and economic manufacturing of surgical guides for oral implant placement. Therefore, the cytotoxicity of a biocopolyester (BE) and a polypropylene (PP), intended for the fused filament fabrication of surgical guides was evaluated. For comparison, a medically certified resin based on methacrylic esters (ME) was printed by stereolithography (n = 18 each group). Human gingival keratinocytes (HGK) were exposed to eluates of the tested materials and an impedance measurement and a tetrazolium assay (MTT) were performed. Modulations in gene expression were analyzed by quantitative PCR. One-way ANOVA with post-hoc Tukey tests were applied. None of the materials exceeded the threshold for cytotoxicity (< 70% viability in MTT) according to ISO 10993-5:2009. The impedance-based cell indices for PP and BE, reflecting cell proliferation, showed little deviations from the control, while ME caused a reduction of up to 45% after 72 h. PCR analysis after 72 h revealed only marginal modulations caused by BE while PP induced a down-regulation of genes encoding for inflammation and apoptosis (*p* < 0.05). In contrast, the 72 h ME eluate caused an up-regulation of these genes (*p* < 0.01). All evaluated materials can be considered biocompatible in vitro for short-term application. However, long-term contact to ME might induce (pro-)apoptotic/(pro-)inflammatory responses in HGK.

## Introduction

Prosthetic backward planning in combination with guided implant placement as available in a digital workflow, minimizes the risk of complications and leads to a predictable rehabilitation^1–3^. Surgical guides are predominantly created by additive manufacturing (AM), as this results in customized and thus convenient aids accompagnied by less material wastage and lower production costs compared to the subtractive fabrication e.g., by milling or grinding^4^. AM of surgical guides is primarily conducted by vat photopolymerization (e.g., stereolithography (SLA) or digital light processing (DLP)), based on light curing of liquid resins^5–7^. Complex and fine-grained objects can be produced by vat photopolymerization, but extensive post-processing procedures such as rinsing, washing and additional light-curing are required to achieve final mechanical properties^6^.

In contrast, material extrusion, also known as fused filament fabrication and under the trademark "fused deposition modeling", is a cost-effective and straightforward three dimensional (3D) printing technology based on the extrusion of a thermoplastic filament through a heated nozzle onto a printing bed^8^. On the one hand, minimal post-processing is required for printed objects by material extrusion, on the other hand, warpage and limited accuracy represent typical limitations of the technique^9–11^. Warpage is a result of residual stress accumulation during the cooling phase and depends in particular on the crystallization degree of the polymer, the thermal properties of the filament, and the printing process^12,13^. However, Pieralli et al. demonstrated in-vitro a similar accuracy for implants inserted with guides by material extrusion and vat photopolymerization^14^. An experimental biocopolyester (BE) with improved temperature resistance was used for the manufacturing of the surgical guides as a cost-effective alternative to resins for vat photopolymerization. Low-crystallization biopolymers are already being used in the non-medical field for material extrusion^14^. Polylactic acids (PLA) and acrylonitrile butadiene styrene polyamide (ABS)^15^ are commonly used for material extrusion, while polyolefins are rarely applied for extrusion-based printing, although they are widely used for plastic production also for medical applications^13^. These high molar mass hydrocarbons are chemically resistant and mechanically durable due to their semi-crystalline properties and are able to undergo steam sterilization as a result of their high Vicat softening temperature^16–18^. Therefore, polyolefins such as polypropylene (PP) may provide a further cost-effective alternative for the production of accurate sterilizable surgical guides with well-balanced property profiles.

When applied in the form of 3D printed surgical guides, polymers come into direct contact with intraoral hard and soft tissue cells. Monomer release was described from resins intended for the vat photopolymerization of surgical guides^19^ and occlusal splints^20^ while several studies mention possible adverse effects of resin-based materials in the oral environment^21–23^. Investigations regarding the potential cytotoxicity of substances eluted from extruded materials on human oral cells are sparse. Therefore, a biological risk assessment of BE and PP prior to their intraoral application is mandatory to evaluate potential cytotoxic effects on human tissue-specific cells.

The present study aimed to evaluate the in vitro biocompatibility of the two processed filaments, BE and PP, by using human gingival keratinocytes (HGK) according to the ISO guidelines 10993-5:2009 ^24^ and 10993-12:2021^25^. As a comparison, a commercially available photopolymerizable resin based on methacrylic-esters (ME) and approved for vat photopolymerization of medical devices (class 1) was used. In order to investigate potential cytotoxic effects, interleukin 1β *(IL1B)*, interleukin 6 *(IL6)* and tumor necrosis factor *(TNF)* encoding for inflammation in HGK were investigated. Genes under study included annexin A5 *(ANAX5)*, Caspase 8 (*CASP8*), and Caspase 9 *(CASP9)* as representatives of (pro-)inflammation in HGK. The null hypothesis assumed no differences in terms of biocompatibility between BE, PP, and ME.

## Material and methods

### Investigational materials

An experimental commercially available biocopolyester filament with a diameter of 1.75 mm (BE; GreenTec Pro, Extrudr, Lauterbach, Austria) based on a polylactic acid-based blend with improved temperature resistance and compostability according to EN 13432:2002^26^ was investigated. In addition, a semi-crystalline polypropylene (PP) filament was extruded from a medical-grade (United States Pharmacopeia (USP) Class VI) injection molding granulate (Healthcare PP, *Purell* type, LyondellBasell Industries B.V., Rotterdam, The Netherlands)^27^ and examined. The PP filament was fabricated using a twin-screw extruder (Teach-Line™, ZK 25 T, Collin, Ebersberg, Germany) at 180 °C and 45 rpm, with a 3.3 mm die diameter, a water-cooling system, and a winding unit (take-off speed 90 mm s^−1^). The resulting filament cross-section exhibited dimensions of 2.8 × 2.6 (± 0.05) mm. The dimensions were determined with a micrometer screw at 50 randomly selected points along the filament. For comparison, a commercially available medical Class I photopolymerizable resin (ISO 10993-1:2009 and USP Class VI) based on methacrylic esters (ME; Dental SG Resin, Formlabs, Boston, MA, USA) and intended for the manufacture of surgical guides by SLA was used. Detailed information regarding the composition of the materials are summarized in Table 1.

**Table 1:** Brand names, manufacturers, and composition according to the manufacturer.

Material	Manufacturer	Matrix
Healthcare PP, *Purell* type	LyondellBasell Industries B.V., Rotterdam, The Netherlands	Unfilled polypropylene impact copolymer, MFI (230 °C/2.16 kg) = 15 g/10 min
GreenTec PRO	Extrudr, Lauterbach, Austria	Biocopolyester blend
Dental SG Resin	Formlabs, Boston, MA, USA	Bisphenol A dimethacrylate; 7,7,9-trimethyl-4,13-dioxo-3,14-dioxa-5,12-diazahexadecane-1,16-diyl bismethacrylate, phenyl bis(2,4,6-trimethylbenzoyl)-phosphine oxide

### Preparation of samples and eluates

A standardized test specimen (15 × 15 × 3 mm) was designed using a Computer-Aided Design (CAD) software (Meshmixer 3.5, Autodesk, Inc., San Rafael, CA, USA) and imported as a standard tessellation language (STL) file into the corresponding nesting software. Thereafter, 18 samples were printed for each of the three groups (PP, BE, and ME) according to ISO 10993-12:2021^25^ which specifies the requirements for sample preparation. Extrusion of both experimental filaments (PP, BE) was performed at a printing speed of 50 mm s^−1^, layer height of 0.2 mm with 100% infill. AM of PP was conducted at a nozzle temperature of 210 °C and a build plate temperature of 60 °C (Ultimaker S5, Ultimaker B.V., The Netherlands). Specimens of group BE were printed at a nozzle temperature of 220 °C and a build plate temperature of 60 °C (Prusa i3, MK3, Prague, Czech Republic). Two different extrusion-based printers were used for the manufacturing of the samples since they were produced at two different locations. Samples of group ME were produced with a layer thickness of 0.05 mm using an SLA printer (Form 2, Formlabs, Boston, MA, USA) and subsequently post-processed with rinsing in 99% isopropanol for 5 min, air drying and light-curing for 30 min at 60 °C (λ = 405 nm). Finally, support structures were removed. Samples were cleaned in 75% ethanol for 5 min and rinsed with water before being processed in a washer-disinfector (PG 8536, Miele, Gütersloh, Germany) containing a liquid detergent (neodisher MediClean forte, Dr. Weigert AG, Zug, Switzerland). All specimens were steam sterilized in an autoclave (Webeco, Series EC, Selmsdorf, Germany) at 134 °C for 5 min. For the preparation of the eluates, each sample was immersed in 5 mL keratinocyte growth medium (KG-M1, PromoCell, Heidelberg, Germany) in six well plates (Greiner Bio-One, Frickenhausen, Germany). One-half of the samples was stored at 37 °C, 97% humidity, and 5% CO_2_ for 24 h and the other half for 72 h. All eluates were kept at 4 °C and absence of light before application to cell cultures.

### Cell cultures and cultivation media

Human gingival keratinocytes (HGK) derived from the oral gingiva were used in this study. The Ethics Committee of the University Medical Center Freiburg, Freiburg, Germany approved the collection of tissue samples for the following cell culture experiments (vote no. 411/08) and donors signed an informed consent. All experiments were carried out in accordance with relevant guidelines and regulations. HGK were immortalized with the human papillomavirus type 16 E6/E7 oncogenes^28^ to obtain a stable cell line with high reproducibility, and cultivated in a low-calcium KG-M1 medium (PromoCell) with kanamycin. Standard cell culture conditions (37 °C; 97% humidity; 5% CO_2_) with standard culture flasks up to size T175 (Greiner Bio-One, Frickenhausen, Germany) were used for cultivation. When reaching approximately 80% of confluency, cells were split using trypsin and manual cell scrapers. DMEM3, containing fetal calf serum (FBS, Biochrom AG, Berlin, Germany), was added to stop the enzymatic reaction of trypsin. The cell numbers were determined by an automated Cell Counter (LUNA, Logos Biosystems, Anyang, South Korea). For the conduction of the experiments, HGK were cultured in passages between 36 and 41.

### Impedance measurement

According to the manufacturer´s instructions, the impedance was measured with a real-time cell analyzing system (RTCA) (iCelligence, ACEA Biosciences, Inc., San Diego, CA, USA). Before adding the cells, 100 µl of cell medium containing the eluates (and without for the control) were applied to each of the eight wells of the E-plate E8 of the RTCA system, and a background impedance measurement without HGK was taken. Subsequently, 400 µl medium were applied to each of the wells containing HGK to achieve a density of 1.1 × 10^4^ cells/cm^2^. Plates were incubated with standard cell culture conditions (37 °C; 97% humidity; 5% CO_2_) for 5 d. RTCA iCelligence software (ACEA Biosciences) was used for data acquisition and analysis. Tests were conducted with three biological and two technical replicates for each of the evaluated polymers. The cell index (CI) describes the result of the impedance induced by adherent cells to the electron flow and was normalized at the time point of substance addition.

### Colorimetric tetrazolium assay (MTT)

A quantitative colorimetric assay with tetrazolium salt (3-(4,5-dimethylthiazol-2-yl)-2,5-diphenyltetrazoliumbromid; MTT) (Abcam, Cambridge, UK) was performed in accordance to the ISO guideline 10993-5:2009^24^ to detect the viability of HGK after incubation with the eluates. This assay is based on the reduction of yellow water-soluble MTT in living cells to blue-violet insoluble formazan. To conduct the MTT assay, 200 µl medium containing HGK were applied to each well of a 96 well plate (Greiner Bio-One, Frickenhausen, Germany) to obtain a density of 1.4 × 10^4^ cells/cm^2^. Subsequently, the plates were incubated at 37 °C for 24 h to allow cell adherence. Cell culture medium was then replaced with the one containing the eluates and cells were re-incubated at previously described conditions. After removing the eluate medium, 100 µl MTT solution was added to the cell cultures and incubated for 3 h. Finally, to measure the formazan concentration, a scanning spectrophotometer (Infinite 200 PRO, Tecan, Männedorf, Switzerland) with a wavelength of 590 nm and the compatible data acquisition software were used.

### RNA isolation and quantitative real-time PCR (qRT-PCR)

For gene expression analysis, HGK were seeded with a density of 3.5 × 10^4^ cells/cm^2^ in 24-well plates (Greiner Bio-One, Frickenhausen, Germany). After adding the 24 h and 72 h eluates, cells were incubated for 72 h at formerly described cell culture conditions, and subsequently lysed with RLT Buffer (Qiagen, Venlo, The Netherlands) and dithiothreitol (Biorad, Berkeley, CA, USA). RNA isolation and purification was performed using the RNeasy mini Kit (Qiagen), while for the analysis of RNA integrity and concentration the Experion RNA Analysis Kit (Biorad) was used. First-strand cDNA was synthesized using the RevertAid First Strand cDNA Synthesis Kit (Thermo Fisher Scientific, Waltham, MA, USA). The quantification of the synthesized cDNA was conducted with the Quant-iT PicoGreen dsDNA Reagent Kit (Life Technologies, Carlsbad, California, USA) and the 200 PRO Microplate Reader (Tecan, Crailsheim, Germany). For each polymerase chain reaction, 5 ng cDNA/µl were used for normalization. The qRT-PCR analysis was performed with the RT2 SYBR Green qPCR Mastermix (SABiosciences, Qiagen) on the CFX96 Real-Time PCR Detection System (Biorad) according to the manufacturer's instructions. Commercially available and pre-validated primers (SABiosciences, Qiagen) were used for the expression analysis of HGK relevant genes (Tables 2 and 3). In addition, the unmodulated housekeeping genes glyceraldehyde-3-phosphate dehydrogenase *(GAPDH)*, ribosomal protein L13a *(RPL13A)* and ubiquitin C *(UBC)* (all SABiosciences, Qiagen) were used for normalization. The qRT-PCR analysis was performed according to the following temperature profile protocol: 10 min at 95 °C (initial denaturation) followed by 40 cycles of denaturation for 15 s at 95 °C, 30 s at 55 °C (annealing) and 30 s at 72 °C (extension). Relative gene expression levels were calculated using the 2^−ΔΔCT^ method according to Livak and Schmittgen^29^ for each biomarker. Data were relative to the housekeeping genes and normalized to the negative control.

**Table 2:** Markers used in qRT-PCR for gene expression analysis.

Gene	RefSeq (mRNA)	Protein	Function
*ANXA5*	NM_001154	Annexin A5	Apoptosis
*CASP8*	NM_001080124	Caspase 8	Apoptosis
*CASP9*	NM_001229	Caspase 9	Apoptosis
*IL1B*	NM_000576	Interleukin 1β	Inflammation
*IL6*	NM_000600	Interleukin 6	Inflammation
*TNF*	NM_000594	Tumor necrosis factor	Inflammation

**Table 3:** Primer sequences of the investigated genes.

Gene	Primer sequences (forward sequence)
*ANXA5*	GTGGCTCTGATGAAACCCTCTC
*CASP8*	AGAAGAGGGTCATCCTGGGAGA
*CASP9*	GTTTGAGGACCTTCGACCAGCT
*IL1B*	CCACAGACCTTCCAGGAGAATG
*IL6*	AGACAGCCACTCACCTCTTCAG
*TNF*	CTCTTCTGCCTGCTGCACTTTG

### Statistical analysis

For statistical analysis, data were tested for homogeneity of variances (Levene test) and analysis of variance (ANOVA) with post-hoc Tukey HSD tests for comparison between the polymers and the negative control. In addition, statistical differences between the evaluated time points were assessed using the Student´s t-test and calculations were performed with the software SPSS Statistics (v.22.0, IBM Corp., Armkonk, New York, USA). The level of significance was set at α = 0.05.

## Results

### Impedance measurement

**Figure 1:**
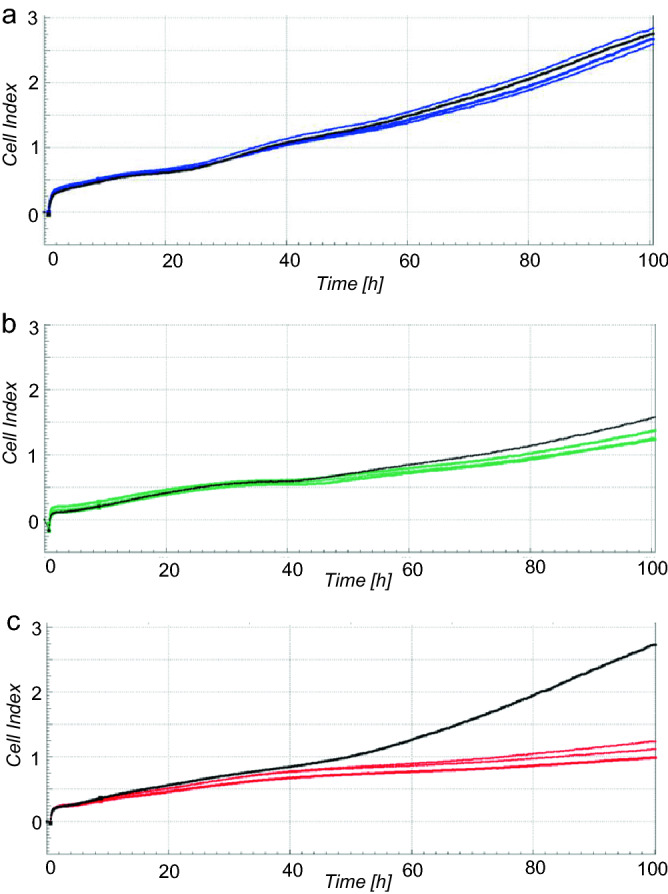
Impedance-based cell index after exposure of HGK to 72 h eluates of PP (**a**), BE (**b**), ME (**c**). The respective non-treated control is visualized in black. Each of the cell indices represents the average of a technical duplicate.

**Figure 2:**
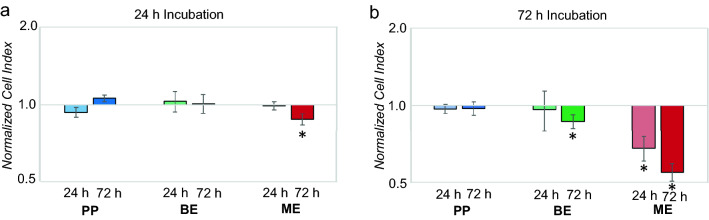
Impedance-based cell index normalized to the non-treated control group after exposure of HGK to eluates of the evaluated polymers (PP, BE, ME) for 24 h (**a**) and 72 h (**b**). The first column of each group represents the cell index after incubation with the 24 h eluate and the second column with the 72 h eluate.

**Figure 3:**
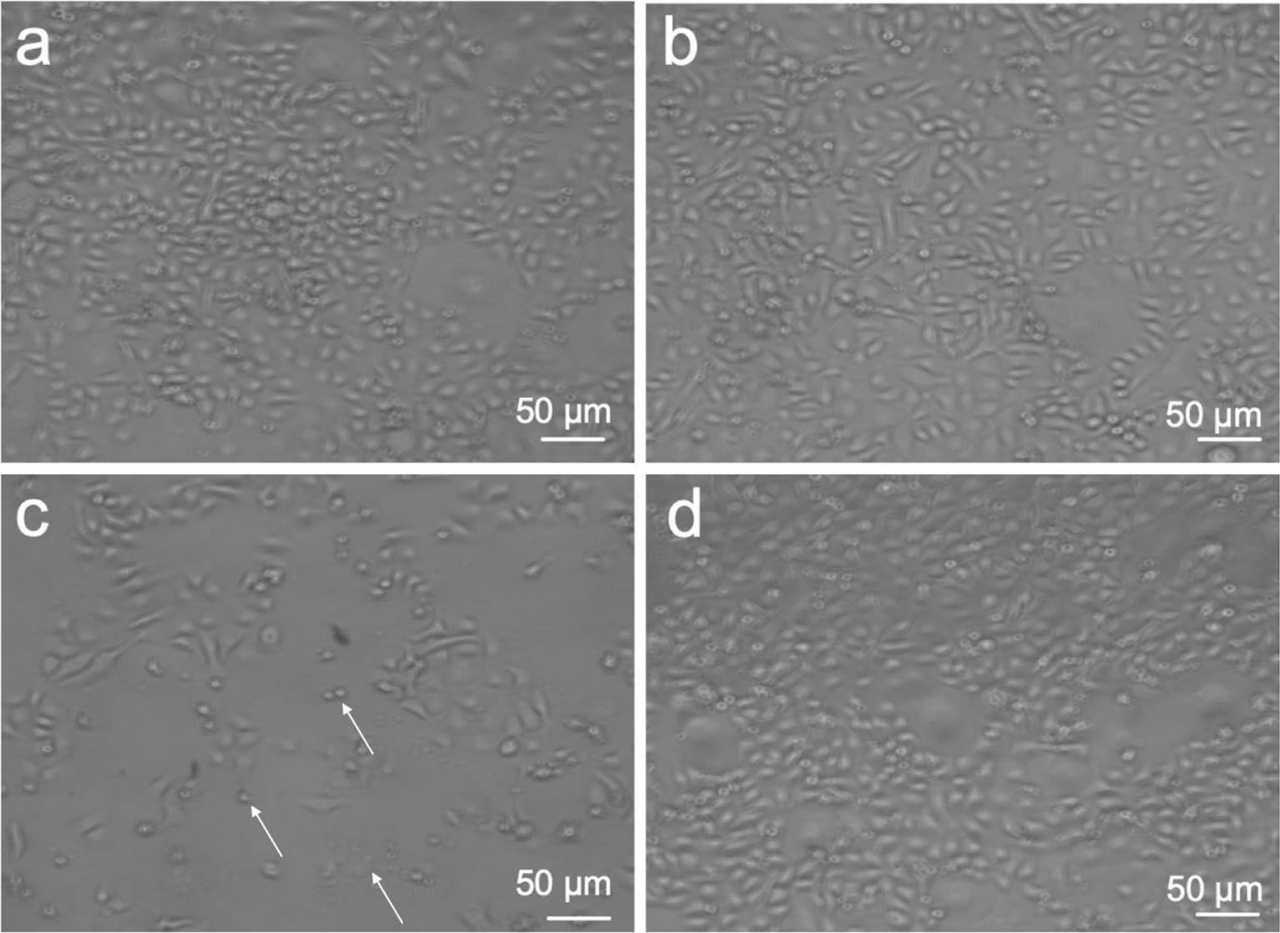
Light microscopic analysis of HGK after incubation with 72 h eluates of PP (**a**), BE (**b**), and ME (**c**) for 72 h in comparison to the non-treated control (**d**). HGK undergoing apoptosis are shown exemplarily using arrows.

A real-time impedance measurement was performed to monitor live cell proliferation, adhesion, and viability of HGK after exposure to the eluates as determined by the CI (Fig. 1). After an incubation period of 24 h, a reduced CI was observed for the 72 h eluates of ME (12% reduction) compared to the untreated control (*p* < 0.05) (Fig. 2a). None of the evaluated 24 h eluates showed a significant regulation of the CI compared to the control after an incubation period of 24 h (*p* > 0.05). After an incubation period of 72 h, both ME eluates (24 h and 72 h eluate) showed a significant inferior CI compared to the control (32% reduction for the 24 h eluate; 45% reduction for the 72 h eluate) (*p* < 0.05) (Figs. 1c, 2b). No significant differences were observed for both PP eluates and the 24 h BE eluate in comparison to the control (*p* > 0.05). However, a reduction of CI of 14% occurred for the 72 h BE eluate compared to the control (*p* < 0.05). The results of the significantly reduced CI after an incubation of 72 h with ME were consistent with the observation under the light microscope (Fig. 3): The cell number was distinctly reduced and the cell morphology was partially apoptotic after incubation with ME (both eluates) for 72 h. In contrast, HGK revealed a confluent cell lawn after incubation with PP and BE for 72 h which had a similar appearance as after incubation with the non-treated control (Fig. 3a,b,d). These observations were consistent with the results of the impedance measurement for PP, L, and the control.

### MTT assay

**Figure 4:**
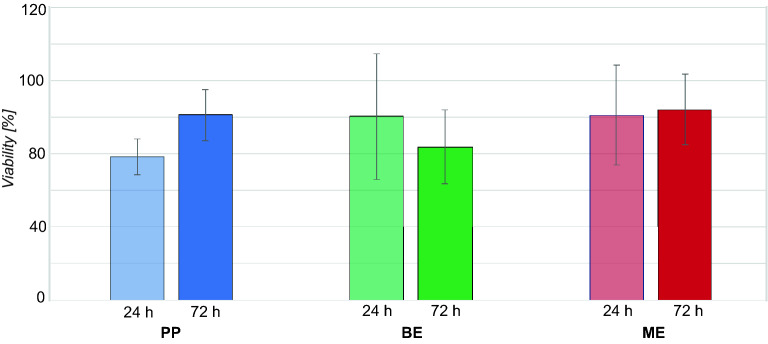
Viability of HGK after exposure to eluates of the tested polymers (PP, BE, ME) in the MTT assay. The first column of each group represents the cell index after incubation with the 24 h eluate and the second column with the 72 h eluate.

The MTT assay showed a viability of 78% after incubation of HGK with the 24 h PP eluate (Fig. 4). The viability was reduced to 84% after incubation with the 72 h BE eluate. Both ME eluates as well as the 72 h PP and the 24 h BE eluate showed no reduced viability in the MTT assay. In accordance with ISO guideline^24^, a test specimen exhibits a cytotoxic potential when the viability is reduced to < 70%, which occurred for none of the evaluated polymers or time points.

### Gene expression analysis

**Figure 5:**
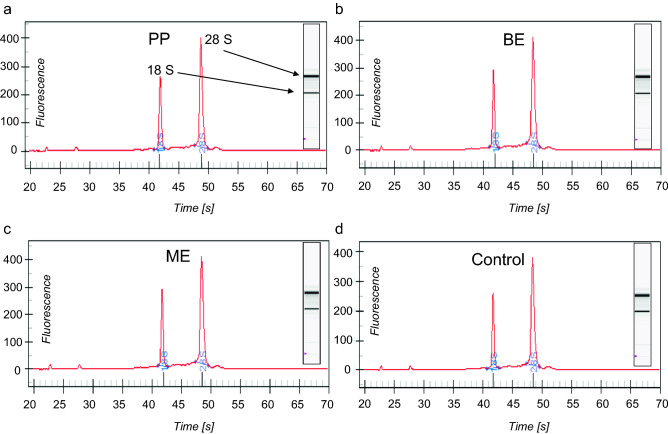
Exemplary electropherograms and virtual gels derived for HGK exposed to PP (**a**), BE (**b**), and ME (**c**) 24 h eluates, as well as unexposed HGK (control, **d**). In the electropherogram, the two peaks depict the 18S and 28S rRNA (arrows in **a**). The virtual gel (inserts in **a**–**d**) is reconstructed based on the peaks and the total RNA concentration.

**Figure 6:**
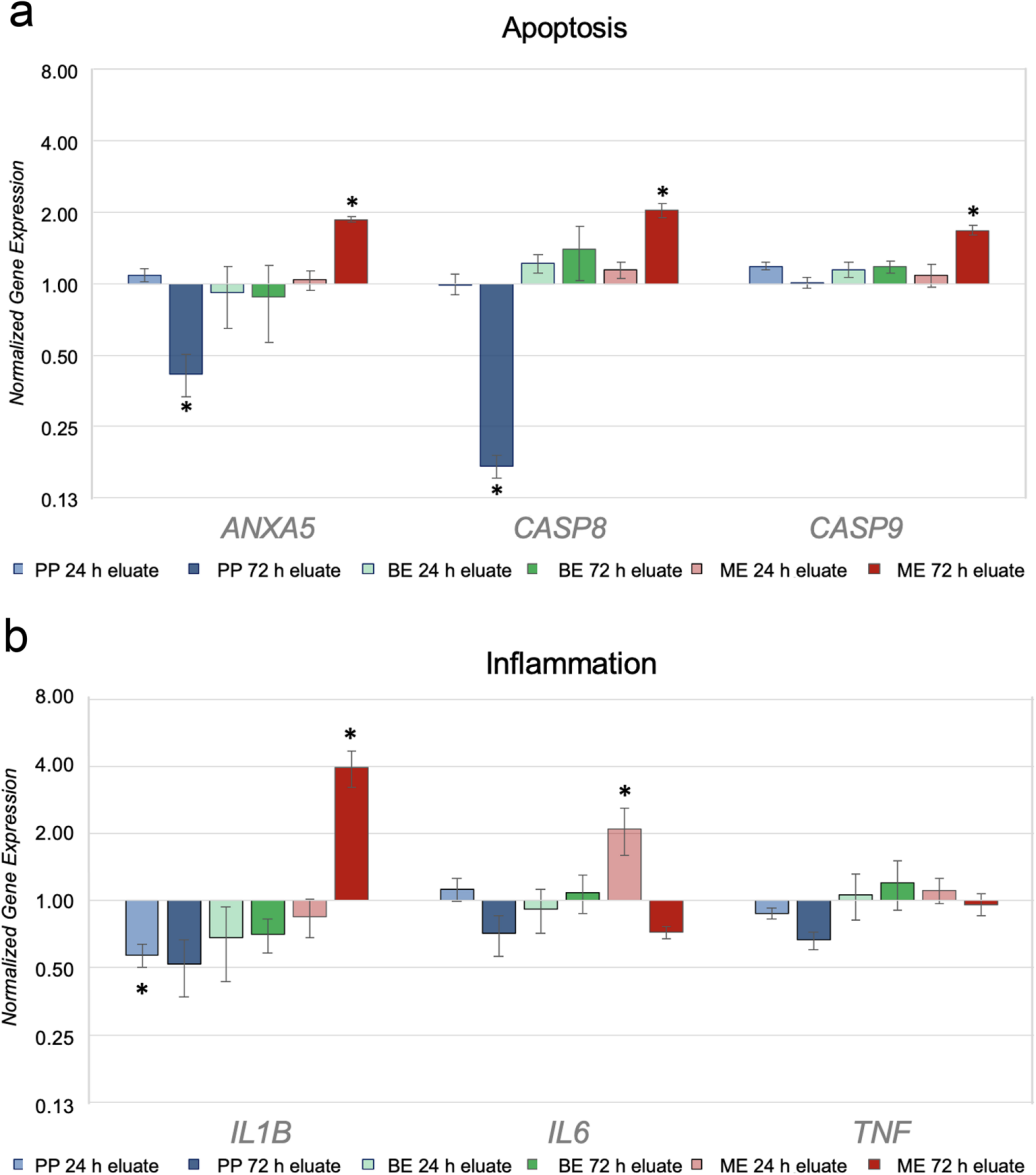
Normalized up-/down-modulation of selected genes of apoptosis (**a**) and inflammation (**b**) in HGK exposed to 24 h and 72 h eluates from PP, BE and ME for 72 h referring to the untreated control. Data are normalized to the untreated control cells and the unregulated housekeeping genes *GAPDH*, *RPL13A* and *UBC*. The normalized expression was calculated using the 2^−ΔΔCT^ method^29^. Asterisks (*) indicate significance (*p* < 0.05) compared to the untreated cells.

A qRT-PCR analysis was performed to investigate possible modulations of relevant genes related to apoptosis and inflammation. Electropherograms and virtual gels (Fig. 5) obtained from the total RNA extraction of HGK, both the unexposed control and after incubation with eluates, showed two distinct peaks for the control 18S and 28S rRNAs. The integrity and amount of total RNA was measured in all groups and considered as appropriate for the qRT-PCR analysis. Figure 6 shows the results of the qRT-PCR analysis for distinct biomarkers related to (pro-)apoptotic and (pro-)inflammatory processes after an incubation period of 72 h.

The biomarkers expression of (pro-)apoptosis *ANXA5*, *CASP8*, and *CASP9* resulted in a significant upregulation after incubation with the ME 72 h eluate compared to the control (*p* < 0.05). In contrast, no significant regulation was observed after incubation with the 24 h eluate (*p* > 0.05). The 24 h eluates of the extrusion-based printed specimens PP and BE showed no up-regulation of the respective biomarkers (*p* > 0.05). After incubation with the 72 h PP eluate, a down-modulation of the biomarkers *ANXA5* and *CASP8* were observed (*p* < 0.05). Comparing the eluate time points for each group, significant differences were found for PP and ME for the biomarkers *ANXA5*, *CASP8*, and *CASP9* (*p* < 0.05). No significant modulations were observed for group BE between the two evaluated eluate time points for all investigated biomarkers (*p* > 0.05).

HGK showed an increase of the inflammatory-related biomarker *IL1B* after incubation with the 72 h ME eluate (*p* = 0.01) compared to the non-treated control. In contrast, the 24 h PP eluate caused a significant down-regulation for *IL1B* (*p* < 0.05). The other tested eluates exhibited a tendency toward down-regulation regarding this inflammation marker. After incubation with the 24 h ME eluate, HGK showed an increased expression of the biomarker interleukin 6 *(IL6)* (*p* < 0.01) in comparison to the untreated control, whereas the 72 h eluate of this polymer caused a tendency toward down-regulation. No significant modulations (*p* > 0.05) compared to the control were observed for *IL6* after incubation with the remaining groups. In addition, the biomarker *TNF* showed no significantly modulated gene expression compared to the control group for the evaluated materials and eluate time points (*p* > 0.05). When comparing 24 h with 72 h eluate time points of group PP, significant differences were observed for the genes *IL6* and *TNF* (*p* < 0.05). Group ME showed significant differences between the two eluate time points for *IL1B* and *IL6* (*p* < 0.05), whereas BE revealed no significant modulations in gene expression of the inflammatory biomarkers between the two eluate times (*p* > 0.05).

## Discussion

Numerous materials for resin-based AM of surgical guides are available on the market but the demand for inexpensive and sustainable materials as well as for simple fabrication workflows is increasing^30^. The objective of the present study was to evaluate the in vitro biocompatibility of two experimental polymers intended for the AM of surgical guides by material extrusion in comparison to a certified light-curable resin used for vat photopolymerization of medical class I surgical guides (ISO 10993-5:2009 and USP Class VI). A commonly used biopolymer for material extrusion in the non-medical field is based on PLA^31^. However, pure PLA is prone to deformation when steam sterilized at temperatures above 121 °C. Therefore, an experimental biocopolyester blend based on PLA, other biopolymers, and calcium carbonate fillers was utilized for optimized temperature resistance. In addition, a semi-crystalline PP was included in the present biocompatibility evaluation. PP belongs to the group of polyolefins which are high-molecular hydrocarbon materials derived from fossil or renewable energy sources^32^. Due to its semi-crystalline nature, PP appears chemically resistant and withstands steam sterilization. A PP injection molding granulate with certified biocompatibility according to USP class VI was used for the extrusion of the PP filament^27^. However, material properties might suffer from alteration during the extrusion or printing process leading to potential cytotoxic effects. The null hypothesis of this study assumed no differences in terms of biocompatibility on HGK between the evaluated materials.

HGK were selected based on their anatomical location on the surface of the oral mucosa. Due to the in-vitro short lifetime of primary HGK with resulting limited reproducibility of the experiments, immortalized HGK were chosen. Nevertheless, the differentiation pattern of this cell line is well-preserved, making HGK comparable to the original tissue and particularly suitable for experiments on modulations of gene expression^28^. The eluates were prepared according to ISO guideline 10993-12:2021, which describes sample preparation and reference materials. Furthermore, the incubation time of 24 h for the MTT assay is defined in ISO standard 10993-5:2009 while the incubation period of 72 h for the qRT-PCR was based on the results of the impedance measurement. The extended incubation period was also used to evaluate materials for long-term intraoral application, such as splints or aligners^33^.

RTCA iCelligence™ and MTT assay were used to measure the proliferation and viability of HGK after incubation with the eluates. None of the eluates showed a reduced viability (threshold set at < 70%) in the MTT assay according to ISO 10993-5:2009, which was in accordance with the RTCA measurement and cells’ morphology after incubation with eluates of both filaments, indicating no cytotoxic effects for PP and L. Solely the 72 h BE eluate showed a slightly reduced CI after several days of incubation. A significantly reduced CI was observed for both ME eluates (24 and 72 h), with HGK also showing a significantly reduced cell density and increased apoptotic appearance under the light microscope. These results did not occur in the MTT assay which might suggest a higher specificity and sensitivity of the RTCA system^34^. A further advantage of the RTCA system is the comprehensive documentation of cellular proliferation, growth, and morphological chances (not only in terms of viability) over the entire period of the investigation, rather than being limited to individual endpoints. In addition, no formazan products are generated as in the MTT assay (which might interact with the compounds of the eluates and affect the final results)^35,36^.

To investigate the influence of the eluates on gene expression level of HGK, a qRT-PCR analysis was performed. Potential cytotoxic effects, which might have led to a reduced CI and morphological changes of HGK after incubation with ME for an extended period, were investigated based on relevant biomarkers for (pro-)apoptosis and (pro-)inflammation after 72 h of incubation. The evaluated caspases *CASP8* and *CASP9* are initiator caspases which can induce apoptosis^37,38^. *ANXA5* binds efficiently to phosphatidylserine and is transported to the outer surface of the plasma membrane in the early apoptosis stage^39^. Furthermore, *IL1B* is secreted from keratinocytes and fibroblasts and represents a marker for the early inflammatory response^40,41^. The presence of *IL6* in the inflamed tissue also plays a decisive role in the onset and maintenance of periodontal inflammatory conditions such as periodontitis and gingivitis^42^. The additionally investigated marker TNF is a pro-inflammatory cytokine associated to tissue degeneration and able to stimulate osteoclastic activity^43^. In the present study, an up-regulation of the early apoptosis biomarkers *ANXA5*, *CASP8* and *CASP9* was observed after incubation with the 72 h ME eluate. Since these modulations did not occur for the 24 h eluate, it can be assumed that the release of cytotoxic compounds occurs after a longer period. Polydorou et al.^44^ showed that light-cured composite materials release monomers over a time period of up to 1 year, which might have adverse effects on oral tissues. Released monomers, such as bisphenol A-glycidyl methacrylate (BisGMA) of a methacrylate-based nanohybrid composite resin contained in a related form in the investigated SLA-resin, induced severe apoptosis in HGK after 4 d^45^. Moreover, in human gingival fibroblasts (HGF) apoptosis induced by oxidative stresses was observed after the release of monomers^22,46^. The tendency toward cytotoxic effects on gene expression level after incubation with the 72 h ME eluates was corroborated with an up-regulation of *IL1B* and *IL6*, with the latter biomarker being regulated by the 24 h eluate. Therefore, the present results suggest that monomers can be released from ME test specimens after an extended period despite elaborated washing and light-curing steps and may induce (pro-)apoptotic/(pro-)inflammatory modulations in HGK. The null hypothesis assuming no differences in cytotoxicity between the evaluated materials was therefore rejected. Resins for vat photopolymerization still contain numerous compounds and photoinitiators in the processed parts that are cytotoxic^47^ and may induce (pro-)apoptotic/(pro-)inflammatory modulations in HGK. This outcome seems in agreement with a similar study, which assessed low cytotoxic effects of the evaluated SLA-resin in mesenchymal stem cells and showed significantly decreased viability in the MTT assay after an extended incubation period, although the printed resin is classified as biocompatible^21^. Zhu et al. questioned the safety of several resins for vat photopolymerization in terms of release of toxic substances in water-based cell culture media^48^. As in our study, cytotoxic effects in form of mortality of aquatic bioindicators were not observed after 24 h but increased dramatically over a prolonged period of up to 72 h^48^. The inconsistencies between official certifications and the observed results might derive from the different incubation periods.

PLA and ABS for extrusion-based AM seem not to induce behavioral abnormalities in zebrafish compared to the evaluated photopolymerizable resins^48^, which is in agreement with our results on gene expression level. None of the extrusion-based materials (PP and BE) showed an up-regulation of the evaluated (pro-)apoptotic/(pro-)inflammatory biomarkers. The significant down-regulation of *ANXA5* and *CASP8* caused by the PP 72 h eluate might even indicate an anti-apoptotic side effect on HGK. These results were enhanced by the activity of the investigated inflammatory markers *IL1B*, *IL6*, and *TNF* which tended toward down-regulation after incubation with PP. These properties are known for polyolefins, which are rarely processed by material extrusion, but are widely used for various medical applications like e.g., tubes, syringes and piezoelectric materials for their high purity and low allergy potential^32,49^. The fact that BE did not show limitations on gene expression level with regard to its biocompatibility is consistent with the literature^50,51^. No adverse side effects in terms of biocompatibility for HGK were observed after extrusion-based AM and steam sterilization. This can be attributed to a different chemical structure and the absence of photoinitiators or monomers in filaments intended for material extrusion, differently than for photo-curable 3D printing materials^19^. Nevertheless, further studies evaluating other cell cultures as e.g. osteoblasts, are needed. An assessment of cells in the tissue compound, such as by means of interactive cell systems of HGK and HGF, would be necessary to mimic similar in-vivo conditions^28^.

## Conclusions

According to the results of this study, all evaluated materials can be considered as biocompatible in vitro for short-term intraoral usage as for guided implant positioning. Furthermore, both experimental filaments showed reduced (pro-)apoptotic/(pro-)inflammatory modulations compared to the investigated SLA-resin. Material extrusion of surgical guides made of PP and BE thus might represent a cost-effective alternative to currently used materials and manufacturing processes.

## Data Availability

All data generated or analysed during this study are included in this published article.
